# Viral Hepatitis in South Korea

**DOI:** 10.5005/jp-journals-10018-1240

**Published:** 2017-09-29

**Authors:** Stella C Pak, Yaseen Alastal, Zubair Khan, Umar Darr

**Affiliations:** 1 Department of Medicine, University of Toledo Medical Center, Toledo, Ohio, USA

**Keywords:** Cirrhosis, Hepatic cellular carcinoma, Hepatitis, South Korea.

## Abstract

In South Korea (S. Korea), viral hepatitis is a major public health burden. Advances in healthcare policy, evidence-based medicine, and therapeutic strategies in S. Korea have brought a rapid change in the sociodemographic and clinical characteristics of viral hepatitis. This review discusses the innovative approaches that S. Korea has taken to curb the epidemic of viral hepatitis. In addition, the efficacy of various preventive and therapeutic modalities is discussed. This review aims to provide a brief overview to guide future research direction and healthcare policy changes.

**How to cite this article:** Pak SC, Alastal Y, Khan Z, Darr U. Viral Hepatitis in South Korea. Euroasian J Hepato-Gastroenterol 2017;7(2):163-165.

## INTRODUCTION

Viral hepatitis is a major public health problem in S. Korea. It is estimated that the economic burden of viral hepatitis in S. Korea is approximately 744.1 million dollars per year.^1^ Improvement of disease control strategies and tools is urgently needed to reduce the economic impact of viral hepatitis. Advances in healthcare policy, evidence-based medicine, and therapeutic strategies in S. Korea have brought a rapid change in the sociodemographic and clinical characteristics of viral hepatitis. In this review, epidemiological and clinical features of infection caused by hepatitis A virus (HAV), hepatitis B virus (HBV), hepatitis C virus (HCV), hepatitis D virus (HDV), and hepatitis E virus (HEV) will be presented.

### Hepatitis A

The total incidence of HAV infection in S. Korea is 62.2 cases per 100, 000 people. Approximately 71.3% of HAV infection occurs in the age group of 20 to 39 years.^2^ The incidence is relatively high compared with the overall hepatitis A incidence in the United States (0.4 cases per 100, 000 people).^3^ The extensive and diverse social activities associated with early adulthood could be attributable for this high prevalence of HAV infection in young adults. Patients with HAV infection usually report recent overseas travel to endemic areas, ingestion of seafood, and catered food service.^4^ Due to improved level of sanitation in S. Korea over past few decades, the incidence of HAV infection in children is relatively low at 11.4 to 12.8 individuals per 100,000 people in the age group under 10. The incidence for adolescents ranges from 26.1 to 49.7 individuals per 100,000 people.^2^ Antibody seroprevalence rate for HAV is about 8% in the age group of 10 to 19 years, suggesting low rate of exposure in childhood and adolescence.^5^ The HAV infection is mostly self-limited and mild, manifesting as anorexia, nausea, vomiting, abdominal discomfort, and jaundice. On the contrary, HAV infection can lead to fulminant hepatitis in adult patients. The reported rate of fulminant hepatitis worldwide is 0.1 to 0.3%. However, the rate of fulminant hepatitis in S. Korea is estimated to range from 0.67 to 1.4%.^1,2,5^ In addition, about 2.7 and 0.47% of acute HAV infection result in acute kidney failure and death respectively.^6^ The high mortality and incidence of fulminant hepatitis and renal complication are likely to be due to low antibody seroprevalence rate and high incidence of HAV infection in adult groups.

In response to this high incidence of HAV infection, the Korean Centers for Disease Control and Prevention added HAV vaccination to the national immunization requirement.^2^ An interesting area for future research would be the impact of national HAV vaccination on the rate of fulminant hepatitis development.

### Hepatitis B

South Korea is an intermediate endemic area for HBV with estimated prevalence of 2 to 7%. Approximately 50 to 80% of HBV infection occurs through perinatal transmission.^7,8^ Thus, universal HBV vaccination for infants is critical in HBV prevention. With implementation of HBV in perinatal vaccination and advance in therapeutic modalities, the prevalence of HBV seroposi-tivity dropped from 8 to 10% in the 1980s and 1990s to 2 to 7% in 2017.^7,9^

About 55% of liver cirrhosis is related to HBV in S. Korea. The HBV infection is also attributable for about 64 to 70% of hepatocellular carcinoma (HCC), which accounts for 15.3% of all-cancer related deaths.^10,11^ Thus, in 1999, the Korean Government adapted HCC screening for patients with chronic hepatitis aged over 40 and individuals with liver cirrhosis. The screening tests consisted of 6-month interval alpha-fetoprotein testing and abdominal ultrasonography. These proactive screening led to improvement of the 5-year survival rate for HCC from 13.2% in 1996 to 2000 to 23.3% in 2003 to 2008.^11^

### Hepatitis C

The HCV seropositivity among Koreans is estimated to be about 0.78%, with the peak age interval being 30 to 49 years. For individuals with HCC, the prevalence of HCV seropositivity ranges from 10 to 15%.^12,13^ About 10% of cirrhosis and 12 to 17% of HCC develop as long-term complication of HCV infection.^10^ Approximately one-third of patients with HCV received treatment with pegylated interferon (pegIFN) in 2015. About 58.3 to 74.7% of patients on pegIFN therapy achieve the sustained virology response (SVR).^14^ The HCV results in cirrhosis or HCC in 20% of infected individuals.^13^ About 75 to 85% of HCV infection develops into chronic hepa-titis.^15^ The high rate of complication might be due to the fact that 65.1% of infected individuals are not aware of HCV infection.^16^ Providing screening to population at risk, including those using IV drugs, living with HCV carrier, having needle stick injury, being on dialysis, or those who received transfusion before 1995 might allow early diagnosis and intervention and, thus, reduce the rate of complication.^13^

Approximately 3% of Koreans are infected with both HBV and HCV. The prevalence of thyroid dysfunction and diabetes mellitus has been reported to be higher than the general population in S. Korea: 6.0 *vs* 3.83% for thyroid dysfunction and 14.7 *vs* 6.1 to 6.9% for diabetes mellitus.^13,16,17^ However, the pathophysiological connection between these conditions and HCV infection remains unknown. Unlike HBV, HCV infection is not a reportable disease in S. Korea; thus, no national database exists for HCV.^16^ Large-scale prospective cohort studies are needed to improve the understanding of HCV and its long-term complications.

With IFN-based therapy in 2015, about 54.6% of patients were not being treated, mostly due to advanced age over 75 years, advanced liver disease, severe renal impairment, IFN-related side effects, and financial difficulty.^14^ Established side effects of IFN include flu-like symptoms, depression, morbilliform drug eruption, dysgeusia, and pancytopenia.^18^ More than 95% of patients on IFN reported having experienced at least one side effect.^19^

As the era of direct-acting antivirals (DAAs) began in early 2010s, 41.7 to 25.3% of patients who could not achieve SVR with IFN therapy now have this alternative therapeutic regimen. Reported adverse effects from DAA are rash, flu-like symptoms, fatigue, diarrhea, and anemia; however, these side effects tend to be milder and less frequent compared with IFN therapy.^19^ The DAA is also shown to be more effective with SVR achievement rate approaching 95%.^20^ The cost for DAA is about $150 to 200 per month compared with $300 to 400 for IFN in S. Korea. The lower cost would help increase access to anti-HCV therapy.^14^ With this newly available therapy, we expect to see an increase in the percentage of patients being treated for chronic hepatitis from HCV. This increase in treatment rate will improve the health status and survival rate in patients with HCV.

### Hepatitis D

The prevalence of HDV infection in S. Korea is estimated to be 0 to 3.6%. This low prevalence suggests that the risk of HDV infection for individuals with HBV in S. Korea is relatively low.^21^ About 3.09% of individuals infected with both HBV and HDV were shown to have HCC.^22^ The possible association between coinfection by HBV and HDV and HCC is a future area of research.

### Hepatitis E

The HEV is not endemic in S. Korea and is responsible for only 2% of acute viral hepatitis.^23^ Surprisingly, the seroprevalence for anti-HEV antibody was about 17 to 34% in Koreans, suggesting a large number of undetected, subclinical HEV infection cases.^23,24^ Further studies are needed to investigate the high seroprevalence of antibodies against HEV in this nonendemic country.

## CONCLUSION

The aim of this review was to provide a brief update on the current status of viral hepatitis in S. Korea. We believe that this review should improve the understanding of the epidemiological status and clinical behavior of viral hepatitis caused by HAV to HEV in this country.
